# Inflammation and endothelial function relevant genetic polymorphisms, carotid atherosclerosis, and vascular events in high-risk stroke population

**DOI:** 10.3389/fneur.2024.1405183

**Published:** 2024-05-16

**Authors:** Hong Chen, Ting Qing, Hua Luo, Ming Yu, Yanfen Wang, Wei Wei, Yong Xie, Xingyang Yi

**Affiliations:** ^1^Department of Neurology, The People’s Hospital of Deyang City, Deyang, Sichuan, China; ^2^Department of Neurology, The Second People’s Hospital of Deyang City, Deyang, China; ^3^Department of Neurology, The Affiliated Hospital of Southwest Medical University, Luzhou, Sichuan, China; ^4^Department of Neurology, The Suining Central Hospital, Suining, Sichuan, China

**Keywords:** carotid atherosclerosis, high-risk stroke population, outcome, inflammation, endothelial function, genetic polymorphism

## Abstract

**Aim:**

To identify the associations of 19 single nucleotide polymorphisms (SNPs) in genes involved in inflammation and endothelial function and carotid atherosclerosis with subsequent ischemic stroke and other vascular events in the high-risk stroke population.

**Methods:**

This was a multicenter community-based sectional survey and prospective cohort study in Sichuan, southwestern China. Eight communities were randomly selected, and the residents in each community were surveyed using a structured face-to-face questionnaire. Carotid ultrasonography and DNA information were obtained from 2,377 out of 2,893 individuals belonging to a high-risk stroke population. Genotypes of the 19 SNPs in genes involved in inflammation and endothelial function were measured. All the 2,377 subjects were followed up for 4.7 years after the face-to-face survey. The primary outcome was ischemic stroke, and the secondary outcome was a composite of vascular events.

**Results:**

Among the 2,377 subjects, 2,205 (92.8%) completed a 4.7-year follow-up, 947 (42.9%) had carotid atherosclerosis [372 (16.9%) carotid vulnerable plaque, 405 (18.4%) mean IMT > 0.9 mm, 285 (12.0%) carotid stenosis ≥15%]. Outcomes occurred in 158 (7.2%) subjects [92 (4.2%) ischemic stroke, 17 (0.8%) hemorrhagic stroke, 48 (2.2%) myocardial infarction, and 26 (1.2%) death] during follow-up. There was a significant gene–gene interaction among *ITGA2* rs1991013, *IL1A* rs1609682, and *HABP2* rs7923349 in the 19 SNPs. The multivariate logistic regression model revealed that carotid atherosclerosis and the high-risk interactive genotypes among the three SNPs were independent with a higher risk for ischemic stroke (OR = 2.67, 95% CI: 1.52–6.78, *p* = 0.004; and OR = 3.11, 95% CI: 2.12–9.27, *p* < 0.001, respectively) and composite vascular events (OR = 3.04, 95% CI: 1.46–6.35, *p* < 0.001; and OR = 3.23, 95% CI: 1.97–8.52, *p* < 0.001, respectively).

**Conclusion:**

The prevalence of carotid atherosclerosis was shown to be very high in the high-risk stroke population. Specific SNPs, interactions among them, and carotid atherosclerosis were independently associated with a higher risk of ischemic stroke and other vascular events.

## Introduction

Stroke is one of the leading causes of adult disability and mortality in the world (1). The incidence of stroke is still increasing at an annual rate of 8.7%, and there are approximately 3 million new stroke patients every year in China (2, 3). Stroke is a multifactorial complex disease, and its pathogenesis remains unclear. Therefore, it is imperative to understand its potential etiology and pathogenesis to prevent stroke and other vascular events more effectively.

Stroke is considered a heterogeneous and multifactorial disease resulting from a combination of genetic risk and environmental factors (4). Carotid atherosclerosis, as an important etiology and risk factor for stroke, is associated with a higher risk of stroke, other vascular events, and cardiovascular mortality as a result of plaque rupture or luminal stenosis (5–7). Carotid atherosclerosis, including carotid plaque, carotid stenosis, and increased intima-media thickness (IMT), is a powerful subclinical predictor for future stroke and other vascular events (7, 8). Although many traditional vascular risk factors are associated with carotid atherosclerosis, several studies have focused their investigation on the effect of genetic etiologies because of their potential contribution to vascular injury (9, 10).

Atherosclerosis is a chronic inflammatory disease where the activation of various inflammatory cells, smooth muscle cell proliferation, and endothelial cell damage play key roles in the pathological mechanisms of atherosclerosis (11, 12). Many studies have revealed that variants in genes related to inflammation and endothelial function are associated with carotid atherosclerosis (10, 13–16). For instance, Gardener et al. (13) evaluated the associations between carotid plaque and 197 variants in 43 genes implicated in inflammation and endothelial function and demonstrated that the variants in 10 genes (*IL6R*, *TNF*, *NOS2A*, *IL1A*, *TNFSF4*, *ITGA2, PPARA*, *TLR4*, *HABP2*, and *VCAM1*) were associated with carotid plaque in the Northern Manhattan population. Similarly, our previous studies have also shown that specific single nucleotide polymorphisms (SNPs) in genes related to inflammation and endothelial function and high-risk interaction among *HABP2* rs7923349, *ITGA2* rs1991013, *IL1A* rs1609682, and *NOS2A* rs8081248, the interaction between ITGA2 rs4865756 and *HABP2* rs7923349, and interaction among *ITGA2* rs1991013, *IL1A* rs1609682, and *HABP2* rs7923349 were associated with carotid plaque vulnerability (10), carotid stenosis and IMT (14, 15), and increased risk of carotid atherosclerosis (16), respectively. However, Gardener et al. and our previous studies did not prospectively investigate the association of these variants and carotid atherosclerosis with subsequent stroke and other vascular events. Information is lacking about the association between these variants and the risk of adverse vascular events in the high-risk stroke population during follow-up.

According to the China National Stroke Screening Survey (CNSSS) program (3), we carried out a multicenter community-based sectional survey among the high-risk stroke population to evaluate the associations between the variants in genes related to inflammation and endothelial function with carotid atherosclerosis in Sichuan, southwestern China (16). Based on the survey, we undertook this prospective cohort study to investigate (1) the incidence of new stroke and other vascular events during follow-up in the high-risk stroke population; (2) the associations between 19 SNPs in genes related to inflammation and endothelial function, as well as carotid atherosclerosis, and their potential impact on subsequent stroke and other vascular events. This study aimed to provide novel insights into the genetic mechanism of carotid atherosclerosis and stroke, which could help in the prevention of carotid atherosclerosis, stroke, and other vascular events.

## Materials and methods

### Study population

Based on our previous community-based cross-sectional survey (16), we undertook this multicenter prospective cohort study in Sichuan, southwestern China, from May 2015 to January 2020. This study is a part of the CNSSS, and the study protocol was approved by the Ethics Committee of the Affiliated Hospital of Southwest Medical University, Suining Central Hospital, and the People’s Hospital of Deyang City. Written informed consent was obtained from each participant.

The community-based cross-sectional survey was carried out in eight communities of Sichuan between May 2015 and September 2015. The detailed procedures relating to recruitment of participants, evaluation of risk factors, and definition of high-risk stroke population were described in our previous articles (10, 14–16). Briefly, the eight communities were selected using the cluster randomization method. Residents aged ≥40 years were screened using a structured face-to-face questionnaire. The eight conventional risk factors for stroke (overweight/obesity, smoking, physical inactivity, family history of stroke, diabetes mellitus, hypertension, atrial fibrillation, and dyslipidemia) were assessed. The diagnostic criteria for the eight risk factors and high-risk stroke population have been described in our previous study (17). The exclusion criteria were: (1) residents declined to participate in this study; (2) history of carotid artery stenting or endarterectomy; (3) severe cardiovascular, liver, and renal disease, acute and chronic inflammation, blood system disease, malignant tumors, and immune system diseases; and (4) incomplete information on both ultrasonography and deoxyribonucleic acid (DNA).

**Figure 1:**
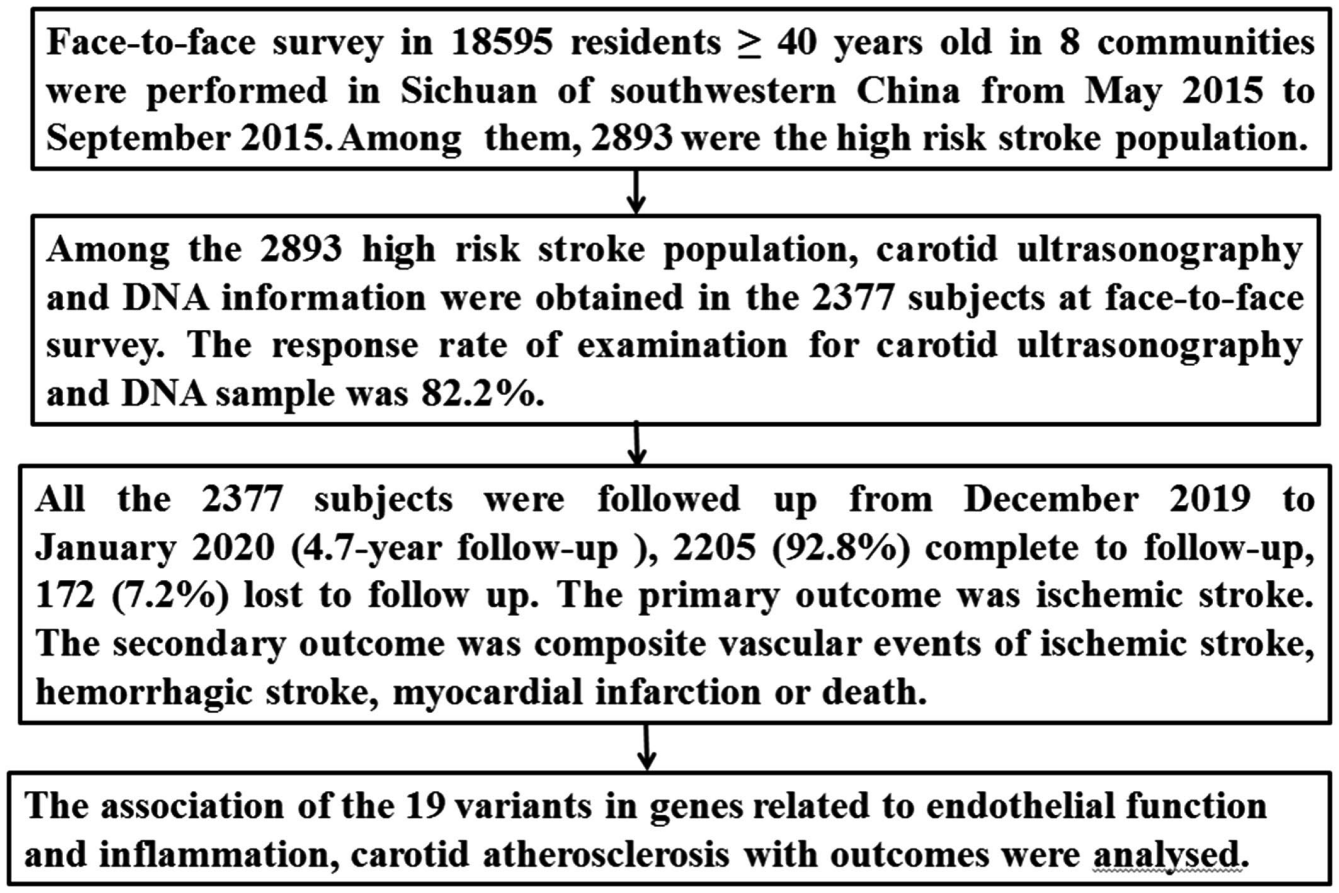
Flow chart of the study.

Of the 16,892 participants in the 8 communities, 2,893 were identified as the high-risk stroke population, from which carotid ultrasonography and DNA information were obtained for 2,377 subjects (Figure 1).

### Carotid ultrasonography

For the high-risk stroke population, bilateral carotid arteries were evaluated by ultrasound investigators using the Color duplex scan (Acuson Sequoia Apparatus, type 512, 7.5-MHz probe, Berlin, Germany) (8, 16). Carotid plaque, IMT, and extracranial carotid stenosis were assessed. The ultrasound investigators were blinded to the clinical data. The detailed procedure and definition for carotid plaques, mean IMT, degree of carotid stenosis, and intraobserver and interobserver coefficients can be found in our previous studies (10, 14–16). Carotid atherosclerosis was defined as mean IMT > 0.9 mm or the presence of any carotid plaque or any carotid stenosis ≥15% (16, 18, 19).

### Genotyping

Nineteen SNPs in 10 genes involved in endothelial function and inflammation were obtained from the NCBI database.^1^ The detailed selection principle for the 19 SNPs has been described in our previous articles (10, 16). In brief, (1) the SNPs were evaluated in our previous studies (10, 14–16); (2) the SNPs have a minor allele frequency > 0.05; (3) the SNPs may induce amino acid changes. The investigators evaluated the genotypes of the 19 SNPs using the matrix-assisted laser desorption/ionization-time of flight mass spectrometry method (10, 16). The investigators were blinded to the clinical data of the subjects.

### Outcomes

All 2,377 subjects from the high-risk stroke population were followed up from December 2019 to January 2020 using telephone interviews or by reviewing their medical charts. The follow-up period was 4.7 years after the face-to-face survey. The primary outcome was ischemic stroke. Ischemic stroke was defined as an acute focal neurological deficit lasting more than 24 h and confirmed by neuroimaging. The secondary outcome included composite vascular events of ischemic stroke, hemorrhagic stroke, myocardial infarction, or death from cardiocerebral vascular events during the follow-up. The evaluators for outcomes were blinded to the 19 SNPs and carotid atherosclerosis. The detailed procedure is presented in Figure 1.

### Statistical analysis

All statistical analyses were performed using the SPSS 17.0 (SPSS Inc., New York, USA). Prevalence of carotid atherosclerosis (including carotid plaque, carotid stenosis, and increased IMT) at the face-to-face survey and the incidence of primary outcome (ischemic stroke) and secondary outcome (composite vascular events) during the follow-up were evaluated. Categorical variables were presented as percentages, and intergroup differences were evaluated by χ^2^ test or Fisher’s exact test. Continuous variables were expressed as mean ± Standard Deviation and intergroup differences were compared by Student *t*-test or analysis of variance if these variables were normally distributed; otherwise, by the Wilcoxon rank-sum test.

Hardy–Weinberg equilibrium for allele frequencies was assessed by χ2-test. Gene–gene interaction among the 19 SNPs was analyzed by a generalized multifactor dimensionality reduction (GMDR) approach (20), as previously described by us (10, 16). A multivariate logistic regression model was used to evaluate independent risk factors for primary and secondary outcomes, and the results were reported as odds ratio (OR) with 95% confidential intervals (CI). The variables that showed a potential association with outcomes (*p* < 0.2) in the univariate analysis were entered into the multivariate logistic regression model. Furthermore, the goodness of fit for the multivariate logistic regression model was evaluated by the Hosmer-Lemeshow (H-L) test, and a *p*-value <0.05 was considered statistically significant.

## Results

### The baseline characteristics of the high-risk stroke population

We enrolled 2,377 subjects from a high-risk stroke population who had both carotid ultrasonography and DNA information (Figure 1). The 2,377 subjects were followed up for 4.7 years after the face-to-face survey; 2,205 (92.8%) completed follow-up, and 172 (7.2%) were lost to follow-up. The baseline characteristics of the 2,205 subjects from the face-to-face survey are presented in Table 1. Among the 2,205 subjects, 1,685 (76.4%) had hypertension, 604 (27.4%) had diabetes mellitus, 709 (32.2%) had dyslipidemia, 766 (34.7) were smoking, and 408 (17.2%) had a history of stroke (321 were ischemic stroke, 87 had hemorrhagic stroke). According to the results of carotid ultrasonography, 947 (42.9%) subjects had carotid atherosclerosis [including 372 (16.9%) carotid vulnerable plaque, 405 (18.4%) mean IMT > 0.9 mm, 285 (12.0%) carotid stenosis ≥15% (50 more than 50% stenosis, 235 15–49% stenosis)] (Table 1).

**Table 1:** Baseline characteristics of high-risk stroke population at face-to-face survey.

Characteristics	*N* = 2,205
Age ≥ 60y (*n*, %)	1,483 (67.3)
Male (*n*, %)	992 (45.0)
Overweight/obesity (*n*, %)	1,206 (54.7)
Smoking (*n*, %)	766 (34.7)
Physical inactivity (*n*, %)	1,411 (64.0)
Hypertension (*n*, %)	1,685 (76.4)
Diabetes (*n*, %)	604 (27.4)
Dyslipidemia (*n*, %)	709 (32.2)
Atrial fibrillation (*n*, %)	46 (2.1)
Family history of stroke (*n*, %)	408 (18.5)
History of ischemic stroke (*n*, %)	321 (14.6)
History of hemorrhagic stroke (*n*, %)	87 (3.9)
Carotid atherosclerosis (*n*, %)	947 (42.9)
Mean IMT ≥ 0.9 mm (*n*, %)	405 (18.4)
Carotid vulnerable plaque (*n*, %)	372 (16.9)
Carotid stable plaque (*n*, %)	418 (19.0)
15–49% carotid stenosis (*n*, %)	235 (10.7)
≥50% carotid stenosis (*n*, %)	50 (2.3)

IMT, intima-media thickness.

### Outcomes

Of the enrolled 2,377 subjects, 2,205 completed a 4.7-year follow-up. Outcomes occurred in 158 (7.2%) subjects, including 92 (4.2%) new ischemic stroke, 17 (0.8%) hemorrhagic stroke, 48 (2.2%) myocardial infarction, and 26 (1.2%) death. Compared with the subjects without outcomes, the subjects with outcomes had a higher proportion of dyslipidemia, atrial fibrillation, history of stroke, carotid atherosclerosis, mean IMT > 0.9 mm, and carotid vulnerable plaque at the face-to-face survey (*p* < 0.05, Table 2).

**Table 2:** Comparison of individuals with and without outcomes.

Characteristics	Patients with outcomes (*n* = 158)	Patients without outcomes (*n* = 2047)	*p*-value
Age ≥ 60y (*n*, %)	115 (72.8)	1,368 (66.8)	0.124
Male (*n*, %)	69 (43.7)	923 (45.1)	0.730
Overweight/obesity (*n*, %)	76 (48.1)	1,130 (55.2)	0.084
Smoking (*n*, %)	58 (36.7)	708 (34.6)	0.589
Physical inactivity (*n*, %)	97 (61.4)	1,314 (64.2)	0.480
Hypertension (*n*, %)	112 (70.9)	1,573 (76.8)	0.089
Diabetes (*n*, %)	59 (30.7)	650 (31.8)	0.147
Dyslipidemia (*n*, %)	66 (41.8)	643 (31.4)	0.007
Atrial fibrillation (*n*, %)	10 (6.3)	36 (1.8)	< 0.001
Family history of stroke (*n*, %)	37 (23.7)	371 (18.1)	0.099
History of ischemic stroke (*n*, %)	43 (27.2)	278 (13.6)	< 0.001
History of hemorrhagic stroke (*n*, %)	22 (13.9)	65 (3.2)	< 0.001
Carotid atherosclerosis (*n*, %)	81 (51.3)	866 (42.3)	0.031
Mean IMT ≥ 0.9 mm (*n*, %)	39 (24.7)	366 (17.9)	0.033
Carotid vulnerable plaque (*n*, %)	43 (27.2)	329 (16.1)	< 0.001
Carotid stable plaque (*n*, %)	28 (17.7)	390 (19.1)	0.681
15–49% carotid stenosis (*n*, %)	23 (14.6)	212 (10.4)	0.099
≥50% carotid stenosis (*n*, %)	4 (2.5)	46 (2.2)	0.999

IMT, intima-media thickness.

According to the results of carotid ultrasonography during the face-to-face survey, the stratified analysis demonstrated that carotid atherosclerosis, mean IMT > 0.9 mm, carotid vulnerable plaque, and mean IMT ≥0.9 mm + vulnerable plaque + ≥ 15% stenosis at the same time were associated with composite vascular events (*p* < 0.05, Table 3). However, we did not find the association between carotid stenosis and outcomes in the present study (*p* > 0.05, Table 2).

**Table 3:** Carotid atherosclerosis and outcomes by stratified analysis (*n*, %).

	Ischemic stroke	Hemorrhagic stroke	Myocardial infarction	Death	Total
**Carotid atherosclerosis**					
Yes (*n* = 947)	47 (5.0)	10 (1.1)	25 (2.6)	12 (1.3)	94 (9.9)
No (*n* = 1,258)	45 (3.6)	7 (0.6)	23 (1.8)	14 (1.1)	89 (7.1)
*p*-value	0.107	0.158	0.193	0.724	0.038
**Mean IMT ≥1.0 mm**					
Yes (*n* = 405)	24 (5.9)	4 (1.0)	13 (3.2)	4 (1.0)	45 (11.1)
No (*n* = 1800)	68 (3.8)	13 (0.7)	35 (1.9)	22 (1.2)	138 (7.7)
*p*-value	0.051	0.812	0.115	0.888	0.023
**Carotid plaque**					
No (*n* = 1,415)	49 (3.5)	8 (0.6)	28 (2.0)	14 (1.0)	99 (7.0)
Stable plaque (*n* = 418)	19 (4.5)	2 (0.5)	7 (1.7)	4 (1.0)	32 (7.7)
Vulnerable plaque (*n* = 372)	24 (6.5)	7 (1.9)	13 (3.5)	8 (2.2)	52 (14.0)
*p*-value	0.034	0.041	0.150	0.189	< 0.001
**Carotid stenosis**					
No (*n* = 1920)	76 (4.0)	14 (0.7)	38 (2.0)	24 (1.3)	152 (7.9)
15–49% stenosis (*n* = 235)	13 (5.8)	3 (1.3)	10 (4.3)	1 (0.4)	27 (11.5)
≥50% stenosis (*n* = 50)	3 (6.0)	0 (0.0)	0 (0.0)	1 (2.0)	4 (8.0)
*p*-value	0.330	0.608	0.066	0.394	0.171
**Mean IMT ≥ 0.9 mm + vulnerable plaque + stenosis**					
Yes (*n* = 89)	7 (7.9)	0 (0.0)	5 (5.6)	1 (1.1)	13 (14.6)
No (*n* = 2,116)	85 (4.5)	17 (0.8)	43 (2.0)	25 (1.2)	170 (8.0)
*p*-value	0.132	0.818	0.057	0.999	0.028

IMT, intima-media thickness.

### Distribution of genotypes in the subjects

The genotype distributions of the 19 SNPs were in agreement with the Hardy–Weinberg Equilibrium (*p* > 0.05). Univariate analyses revealed there were significant differences in genotype distribution of *PPARA* rs4253655, *IL1A* rs1609682, and *HABP2* rs7923349 between subjects with and without carotid atherosclerosis (*p* < 0.05, Table 4). However, we did not find significant differences in genotype distribution of the 19 SNPs between subjects with and without outcomes (*p* > 0.05, Table 4).

**Table 4:** Genotype distribution in individuals with and without carotid atherosclerosis and with and without outcomes (%).

	Carotid atherosclerosis			Outcomes		
	Yes (*n* = 947)	No (*n* = 1,258)	*p*-value	Yes (*n* = 158)	No (*n* = 2047)	*p*-value
*IL6R* (rs4845625)			0.105			0.156
TT	272 (28.7)	318 (25.3)		49 (31.0)	541 (26.4)	
CC	207 (21.9)	313 (24.9)		28 (17.7)	492 (24.0)	
CT	468 (49.4)	627 (49.8)		81 (51.3)	61,014 (49.5)	
*IL6R* (rs1386821)			0.306*			0.539*
GT	65 (6.9)	107 (8.5)		16 (10.1)	156 (7.6)	
GG	3 (0.3)	3 (0.2)		0 (0.0)	6 (0.3)	
TT	879 (92.8)	1,148 (91.3)		142 (89.9)	1885 (92.1)	
*IL1A* (rs1800587)			0.528			0.664*
AG	128 (13.5)	164 (13.0)		23 (14.6)	269 (13.1)	
GG	815 (86.1)	1,084 (86.2)		135 (85.4)	1764 (86.2)	
AA	4 (0.4)	10 (0.8)		0 (0.0)	14 (0.7)	
*IL1A* (rs1609682)			0.033			0.526
GG	418 (44.1)	534 (42.4)		75 (47.5)	877 (42.8)	
GT	446 (47.1)	645 (51.3)		72 (45.6)	1,019 (49.8)	
TT	83 (8.8)	79 (6.3)		11 (6.9)	151 (7.4)	
*PPARA* (rs4253778)			0.705*			0.360*
CG	2 (0.2)	4 (0.3)		1 (0.6)	5 (0.2)	
GG	945 (99.8)	1,254 (99.7)		157 (99.4)	2042 (99.8)	
*PPARA* (rs4253655)			0.034*			1.0*
AG	4 (0.4)	0 (0.0)		0 (0.0)	4 (0.2)	
GG	943 (99.6)	1,258 (100.0)		158 (100.0)	2043 (99.8)	
*TLR4* (rs752998)			0.235			0.103*
TT	17 (1.8)	32 (2.5)		0 (0.0)	49 (2.4)	
GG	680 (71.8)	867 (68.9)		112 (70.9)	1,435 (70.1)	
GT	250 (26.4)	359 (28.5)		46 (29.1)	563 (27.5)	
*TLR4* (rs1927911)			0.832			0.059
AG	468 (49.4)	611 (48.6)		90 (57.0)	984 (48.1)	
AA	143 (15.1)	185 (14.7)		16 (10.1)	317 (15.5)	
GG	336 (35.5)	462 (36.7)		52 (32.9)	746 (36.4)	
*TNFSF4* (rs1234313)			0.065			0.249
AG	453 (47.8)	539 (42.8)		81 (51.3)	911 (44.5)	
GG	104 (11.0)	148 (11.8)		15 (9.5)	237 (11.6)	
AA	390 (41.2)	571 (45.4)		62 (39.2)	899 (43.9)	
*TNFSF4* (rs11811788)			0.853			0.522*
CG	145 (15.3)	199 (15.8)		20 (12.6)	324 (15.8)	
GG	11 (1.2)	12 (1.0)		2 (1.3)	21 (1.0)	
CC	791 (83.5)	1,047 (83.2)		136 (86.1)	1702 (83.1)	
*NOS2A* (rs8081248)			0.987			0.864
AG	420 (44.3)	560 (44.6)		68 (43.0)	912 (44.6)	
AA	101 (10.7)	136 (10.8)		16 (10.1)	221 (10.8)	
GG	426 (45.0)	562 (44.7)		74 (46.8)	914 (44.7)	
*NOS2A* (rs2297518)			0.973			0.798
AG	253 (26.7)	341 (27.1)		45 (28.5)	549 (26.8)	
AA	19 (2.0)	26 (2.1)		4 (2.5)	41 (2.0)	
GG	675 (71.3)	891 (70.8)		109 (69.0)	1,457 (71.2)	
*TNF* (rs3093662)			0.341			0.763
AG	48 (5.1)	53 (4.2)		8 (5.1)	93 (4.5)	
AA	899 (94.9)	1,205 (95.8)		150 (94.9)	1954 (95.5)	
*VCAM1* (rs2392221)			0.122			0.414
CT	236 (24.9)	269 (21.4)		32 (20.2)	473 (23.1)	
CC	691 (73.0)	956 (76.0)		124 (78.5)	1,523 (74.4)	
TT	20 (2.1)	33 (2.6)		2 (1.3)	51 (2.5)	
*VCAM1* (rs3783615)			-			-
AA	947 (100.0)	1,258 (100.0)		158 (100.0)	2047 (100.0)	
*HABP2* (rs7923349)			0.002			0.280
TT	64 (6.8)	47 (3.7)		5 (3.2)	106 (5.2)	
GT	365 (38.5)	462 (36.7)		67 (42.4)	760 (37.1)	
GG	518 (54.7)	749 (59.5)		86 (54.4)	1,181 (57.7)	
*HABP2* (rs932650)			0.566			0.449
CT	418 (44.1)	543 (43.2)		74 (46.8)	887 (43.3)	
CC	83 (8.8)	127 (10.1)		11 (7.0)	199 (9.7)	
TT	446 (47.1)	588 (46.7)		73 (46.2)	961 (46.9)	
*ITGA2* (rs1991013)			0.208			0.580
GG	77 (8.1)	129 (10.3)		17 (10.8)	189 (9.2)	
AA	440 (46.5)	584 (46.4)		77 (48.7)	947 (46.3)	
AG	430 (45.4)	545 (43.3)		64 (40.5)	911 (44.5)	
*ITGA2* (rs4865756)			0.308			0.286
AG	371 (39.2)	457 (36.3)		65 (41.1)	763 (37.3)	
GG	514 (54.3)	724 (57.6)		80 (50.6)	1,158 (56.6)	
AA	62 (6.5)	77 (6.1)		13 (8.2)	126 (6.1)	

*Fisher’s exact test.

### Gene–gene interaction

The gene–gene high-order interaction among the 19 SNPs was analyzed using the GMDR approach, as previously described by us (16); the results of the gene–gene interaction among the 19 SNPs have been described in our previously published article (16). In brief, there was a significant gene–gene interaction among *ITGA2* rs1991013, *IL1A* rs1609682, and *HABP2* rs7923349 using the GMDR [Tables 3, 4 in our previously published article (16)]. Then, the association of different genotype combinations in the three interactive SNPs with the risk of carotid atherosclerosis was evaluated. Compared with the subjects carrying wild-type genotypes, those subjects carrying rs1991013 AA, rs1609682 TT, and rs7923349 TT (OR = 2.62, 95% CI: 1.21–6.86, *p* = 0.007); rs1991013 AA, rs1609682 GT, and rs7923349 GT (OR = 1.76, 95% CI: 1.03–2.63, *p* = 0.043); rs1991013 AA, rs1609682 TT, and rs7923349 GT (OR = 2.14, 95% CI: 1.18–5.37, *p* = 0.012); and rs1991013 AG, rs1609682 TT, and rs7923349 TT (OR = 2.06, 95% CI: 1.07–4.62, *p* = 0.036) had a higher risk of carotid atherosclerosis, which were defined as high-risk interactive genotypes, otherwise, were considered as the low-risk interactive genotypes (16).

### Association of the high-risk interactive genotypes with carotid atherosclerosis and outcomes

Among the 2,205 subjects, there were 484 subjects carrying the high-risk interactive genotypes. The prevalence of carotid atherosclerosis was higher in the subjects carrying the high-risk interactive genotypes than those carrying the low-risk interactive genotypes (55.2% [267/484] vs. 39.5% [680/1721], χ^2^ = 48.68, *p* < 0.001). Furthermore, the incidence of ischemic stroke and composite vascular events during follow-up was found to be significantly higher in the subjects carrying the high-risk interactive genotypes than those carrying the low-risk interactive genotypes (7.0% [34/484] vs. 3.4% [58/1721], χ^2^ = 12.62, *p* < 0.001, and 11.2% [54/484] vs. 6.0% [104/1721], χ^2^ = 14.85, *p* < 0.001, respectively).

### Multivariable logistic regression analysis of risk factors for outcomes

A multivariate logistic regression model was employed to assess the risk of ischemic stroke and composite vascular events conferred by the high-risk interactive genotypes and carotid atherosclerosis. The high-risk interactive genotypes were defined as one, and the low-risk interactive genotypes were defined as zero. The other variables that exhibited a potential association with outcomes (*p* < 0.2) in the univariate analysis were entered into the regression model. The results revealed that a history of stroke (OR = 2.38, 95% CI: 1.56–6.26, *p* = 0.007), carotid atherosclerosis (OR = 2.67, 95% CI: 1.52–6.78, *p* = 0.004), vulnerable plaque (OR = 2.24, 95% CI: 1.32–6.23, *p* = 0.005), mean IMT ≥0.9 mm + vulnerable plaque + stenosis at the same time (OR = 3.02, 95% CI: 1.97–8.82, *p* < 0.001), *IL6R* rs4845625TT (OR = 1.01, 95% CI: 1.02–3.24, *p* = 0.046), and high-risk interactive genotypes (OR = 3.11, 95% CI: 2.12–9.27, *p* < 0.001) were independent risk factors for ischemic stroke (Table 5), and the goodness of fit of this regression model was well by the H-L test (χ^2^ value =3.03，*p* = 0.823). Furthermore, we found that a history of stroke (OR = 3.01, 95% CI: 1.85–7.96, *p* = 0.005), carotid atherosclerosis (OR = 3.04, 95% CI: 1.46–6.35, *p* < 0.001), mean IMT ≥0.9 mm (OR = 1.89, 95% CI: 1.02–2.64, *p* = 0.037), vulnerable plaque (OR = 2.42, 95% CI: 1.26–6.07, *p* = 0.006), carotid IMT ≥0.9 mm + vulnerable plaque + stenosis at same time (OR = 3.32, 95% CI: 2.13–8.64, *p* < 0.001), *IL6R* rs4845625TT (OR = 1.03, 95% CI: 1.04–2.63, *p* = 0.041), and high-risk interactive genotypes (OR = 3.23, 95% CI: 1.97–8.52, *p* < 0.001) were independent risk factors for composite vascular events (Table 6), and the goodness of fit of this model was well by the H-L test (χ^2^ value =3.36, *p* = 0.912).

**Table 5:** Multivariable regression analysis of risk factors for ischemic stroke.

Factor	OR	95% CI	*p*-value
Age ≥ 60 years	0.99	0.78–1.72	0.322
Overweight/obesity	0.97	0.82–1.32	0.287
Hypertension	1.17	0.97–3.57	0.137
Diabetes	0.92	0.63–1.82	0.362
Dyslipidemia	1.24	0.84–3.02	0.467
Atrial fibrillation	1.36	0.95–2.74	0.102
Family history of stroke	1.81	0.83–3.24	0.231
History of stroke	2.38	1.56–6.26	0.007
Carotid atherosclerosis	2.67	1.52–6.78	0.004
Mean IMT ≥0.9 mm	1.42	0.92–2.36	0.142
Vulnerable plaque	2.24	1.32–6.23	0.005
≥ 15% carotid stenosis	1.01	0.97–2.88	0.207
Mean IMT ≥ 0.9 mm + vulnerable plaque + stenosis at same time	3.02	1.97–8.82	<0.001
*IL6R* rs4845625TT	1.01	1.02–3.24	0.046
*TLR4* rs752998GT	0.93	0.83–2.87	0.285
*TLR4* rs1927911AG	1.13	0.97–3.82	0.213
High-risk interactive genotypes	3.11	2.12–9.27	<0.001

OR, odds ratio; CI, confidence interval; IMT, intima-media thickness.

**Table 6:** Multivariable regression analysis of risk factors for composite vascular events.

Factor	OR	95% CI	*p*-value
Age ≥ 60 years	1.04	0.77–1.62	0.235
Overweight/obesity	0.94	0.88–1.21	0.362
Hypertension	1.24	0.91–3.48	0.092
Diabetes	0.96	0.78–1.76	0.284
Dyslipidemia	1.54	0.96–2.87	0.422
Atrial fibrillation	1.45	0.89–2.86	0.136
Family history of stroke	2.02	0.95–4.47	0.132
History of stroke	3.01	1.85–7.96	0.005
Carotid atherosclerosis	3.04	1.46–6.35	<0.001
Mean IMT ≥ 0.9 mm	1.89	1.02–2.64	0.037
Vulnerable plaque	2.42	1.26–6.07	0.006
≥ 15% carotid stenosis	1.25	0.94–3.21	0.117
Mean IMT ≥ 0.9 mm + vulnerable plaque + stenosis at same time	3.32	2.13–8.64	<0.001
*IL6R* rs4845625TT	1.03	1.04–2.63	0.041
*TLR4* rs752998 GT	1.12	0.98–2.67	0.239
*TLR4* rs1927911AG	1.01	0.93–2.02	0.265
High-risk interactive genotypes	3.23	1.97–8.52	<0.001

OR, odds ratio; CI, confidence interval; IMT, intima-media thickness.

## Discussion

In this study, we observed that the prevalence of carotid atherosclerosis was extremely high in the high-risk stroke population. Carotid atherosclerosis was independently associated with a higher risk for ischemic stroke and composite vascular events during follow-up. Furthermore, we identified the specific SNPs and interaction among *IL1A* rs1609682, *ITGA2* rs1991013, and *HABP2* rs7923349 in genes involved in inflammation and endothelial function increased the risk of carotid atherosclerosis, ischemic stroke, and composite vascular events.

Several studies have revealed that carotid atherosclerosis (including carotid plaque, increased IMT, and carotid stenosis) is associated with a higher risk of stroke and other vascular events (5–7) and is thought to be a powerful subclinical predictor for future vascular events (7, 8). In the current study, we found that carotid atherosclerosis, mean IMT > 0.9 mm, and carotid vulnerable plaque increased the risk of subsequent ischemic stroke and composite vascular events in the high-risk stroke population. Carotid IMT is widely used as an intermediate phenotype for atherosclerosis (15). Previous studies of carotid atherosclerosis have demonstrated that increased IMT may predict future vascular events independently of conventional risk factors (21–23). Carotid plaque burden is an important subclinical precursor of stroke, coronary events, and cardiovascular mortality (7, 8, 10). Certain plaque phenotypes, such as irregular plaques, echolucent or ulcerative plaques, and maximal carotid plaque thickness, may be important markers of vulnerable plaques susceptible to rupture or luminal stenosis leading to stroke or vascular events (8, 24). Carotid stenosis is usually accompanied by carotid-vulnerable plaque and emboli that reduce blood flow to the brain, thereby increasing the risk of stroke as well as other vascular events (14). Asymptomatic carotid stenosis is thought to be an independent predictor of future vascular events (25). However, we did not find an association between carotid stenosis and stroke or other vascular events in this study. The reason may be that the prevalence of carotid stenosis was very low [only 285 (12.0%) had carotid stenosis ≥15%] in this study. It is common knowledge that intracranial stenosis is more prevalent than extracranial stenosis in China. This multicenter community-based sectional survey was a part of the CNSSS program, and intracranial stenosis was not evaluated. Therefore, we did not know the prevalence of intracranial stenosis and its effect on outcomes.

The current study and previous studies have revealed that carotid atherosclerosis increases the risk of vascular events (7, 8, 24, 25). Hence, it is extremely important to investigate the potential etiologies and mechanisms of carotid atherosclerosis for better prevention of stroke and other vascular events. Atherosclerosis is a chronic inflammatory disorder related to various immune-inflammatory cells and inflammatory mediators (11). Activation of various inflammatory cells, smooth muscle cell proliferation, and endothelial cell injury are important mechanisms of atherosclerosis (12). Gardener et al. (13) were the first to investigate the association of 197 variants in 43 genes related to endothelial function and inflammation with a carotid plaque among Hispanics from Northern Manhattan. They found that variants in ten genes linked with inflammation (*NOS2A*, *TNF*, *IL6R*, *PPARA*, *TNFSF4*, *TLR4*, and *IL1A*) and endothelial function (*ITGA2*, *HABP2*, and *VCAM1*) were associated with carotid plaque phenotypes. There were interactions among the haplotypes in *IL6R, TNFSF4, NOS2A,* and *PPARA* for thick plaque and haplotypes in *PPARA* and *IL1A* for irregular plaque. Our previous studies and this study also demonstrated that specific SNPs and high-risk interactions among these SNPs in genes linked with inflammation and endothelial function increased the risk for carotid vulnerable plaque (10), carotid stenosis (14), and carotid atherosclerosis (16).

Although our previous studies and Gardener et al. (13) revealed the association of variants in genes involved in inflammation and endothelial function with carotid atherosclerosis, information about the association between these variants and the risk of stroke and other vascular events was lacking. Therefore, we conducted this multicenter prospective cohort study based on our previous community-based cross-sectional survey (16) and found that *IL6R* rs4845625TT significantly increased ischemic stroke and composite vascular events. Interleukin-6 (IL-6) as a cytokine plays an important role in the “response to injury” model of endothelial cells and atherosclerosis (26), and it is encoded and regulated by the IL-6 receptor (*IL6R*) gene, which is located in the chromosome 1q21. Some studies revealed that polymorphisms of *IL-6R* increased the risk of ischemic stroke in patients with metabolic syndrome and were associated with the neurologic status of ischemic stroke patients (27, 28). However, we did not find the associations between the other 18 SNPs and outcomes in our linkage analysis. Stroke is a heterogeneous and multifactorial complex disease and does not follow the Mendelian pattern of inheritance (4). It may be the result of a gene–gene interaction, and a single genetic variant may not be effective in finding the genetic etiology of stroke (9, 20). Therefore, linkage analysis is not suitable to investigate the genetic etiologies of stroke (20). The noteworthy finding in the current study was that we found a significant gene–gene interaction among *ITGA2* rs1991013, *IL1A* rs1609682, and *HABP2* rs7923349. The high-risk interactive genotypes in the three SNPs independently increased subsequent ischemic stroke and composite vascular events.

The molecular mechanisms of interaction among the three SNPs increasing the risk of subsequent ischemic stroke and other vascular events remain unclear. Integrin alpha 2 (ITGA2) regulates endothelial cell adhesion and cell-surface-mediated signaling. Gardener et al. (13) found that *ITGA2* rs1991013 SNPS increased the risk of carotid plaque and general atherosclerosis. Other studies demonstrated that *ITGA2* C807T SNPs were associated with carotid IMT plaque and had a higher risk of ischemic stroke in patients with type 2 diabetes mellitus (29, 30). Interleukin-1A (IL-1A), as a cytokine, was up-regulated in astrocytes, microglia, and endothelial cells after acute middle cerebral artery occlusion and aggravated cerebral ischemic injury (31). Knockout *IL-1A* gene in mice may reduce ischemic injury (32). Polymorphisms in *IL1A* can increase the risk of ischemic stroke and coronary artery disease (33, 34). *IL1A* allele 2 might influence the inflammatory environment in vascular endothelium and significantly increase the susceptibility of carotid atherosclerosis (35).

The endothelial function maintains the vascular barrier. Endothelial dysfunction may damage vascular integrity and vascular barrier and is associated with various diseases such as atherosclerosis, coronary artery disease, and stroke (36). The hyaluronan-binding protein (HABP2) regulates vascular integrity, and the *HABP2* gene encodes hyaluronan-binding protein. A variant in the *HABP2* gene is a genetic susceptibility locus to stroke (37). Previous studies showed that a variant in *HABP2* was associated with the risk of atherosclerosis (10, 16), venous thromboembolic disease (38), and stroke (37, 39).

Atherosclerosis is one of the important risk factors for stroke; endothelial injury, activation of inflammatory cells, and smooth muscle cell proliferation play key roles in atherosclerosis and stroke (12, 39). Furthermore, maintaining blood–brain barrier integrity is crucial for the homeostasis of the central nervous system. The intense inflammation occurring during the acute phase of ischemic stroke is associated with blood–brain barrier breakdown neuronal injury and plays a critical role in ischemic stroke pathology (40). Several studies have revealed the associations of variants in *IL1A, ITGA2*, *IL-6R,* and *HABP2* with inflammation and endothelial function (13–16, 30). Consequently, a possible explanation for the interaction among the three SNP contributions to a higher risk of carotid atherosclerosis, ischemic stroke, and other vascular events may be that the three SNPs are involved in encoding and regulating the relevant enzymes, which affect the pathogenesis of atherosclerosis and stroke, inflammation and endothelial function, resulting in the occurrence of stroke and other vascular events. However, further studies are needed to investigate the interaction mechanisms among the three SNPs.

This study had several major strengths. First, this multicenter population-based cross-sectional survey and prospective cohort study focused on the high-risk stroke population. Second, we investigated the prevalence of carotid atherosclerosis (including carotid plaque, increased IMT, and carotid stenosis) in the high-risk stroke population for the first time and evaluated the association of carotid atherosclerosis with future ischemic stroke or other vascular events. Third, we used the GMDR to analyze gene–gene interactions among the 19 SNPs and the effect of these gene–gene interactions on future ischemic stroke and other vascular events. Fourth, our study had a long follow-up (4.7 years). However, there were also several limitations in the present study. First, there might be recall bias because of the self-reported questionnaire and follow-up by telephone. Second, according to the CNSSS program, only extracranial carotid arteries were evaluated by ultrasound; intracranial arteries were not examined by high-resolution magnetic resonance imaging, computed tomography, or digital subtraction angiography. Hence, we did not know the information about intracranial atherosclerosis and its effect on outcomes. Third, we only evaluated the role of the 19 known SNPs in genes involved in endothelial function and inflammation, and other relevant SNPs were not investigated. Fourth, although we found that the high-risk interactive genotypes in *ITGA2* rs1991013, *IL1A* rs1609682, and *HABP2* rs7923349 independently increased ischemic stroke and other vascular events, the detailed molecular mechanisms remain unclear. Finally, our previous study has shown that the persistence of drug therapy (including statins, antiplatelet drugs, and antihypertensives) was associated with a lower risk for stroke and other vascular events (41). The major aim of the present study was to investigate the associations of the 19 SNPs and the interaction among these SNPs with outcomes. Therefore, we did not examine the effect of these drugs on outcomes.

## Conclusion

The prevalence of carotid atherosclerosis was shown to be very high in the high-risk stroke population. Carotid atherosclerosis was significantly associated with a higher risk for ischemic stroke and other vascular events. We also identified significant associations of specific SNPs in genes related to inflammation and endothelial function with carotid atherosclerosis and outcomes. Furthermore, we found a significant gene–gene interaction among *ITGA2* rs1991013, *IL1A* rs1609682, and *HABP2* rs7923349, and the high-risk interactive genotypes in the three SNPs independently increased ischemic stroke and other vascular events. These findings are expected to identify novel genetic targets for preventing and treating carotid atherosclerosis and stroke or other vascular events and provide theoretical bases for future gene therapy and drug development against these targets.

## Data availability statement

The original contributions presented in the study are publicly available. This data can be found at: https://datadryad.org/, https://doi.org/10.5061/dryad.k98sf7m9r.

## Ethics statement

The studies involving humans were approved by the Ethics Committee of the Affiliated Hospital of Southwest Medical University, Suining Central Hospital, and the People’s Hospital of Deyang City. The studies were conducted in accordance with the local legislation and institutional requirements. Written informed consent for participation in this study was provided by the participants’ legal guardians/next of kin.

## Author contributions

HC: Funding acquisition, Supervision, Writing – original draft. TQ: Data curation, Formal analysis, Methodology, Writing – review & editing. HL: Visualization, Writing – original draft. MY: Supervision, Validation, Writing – review & editing. YW: Methodology, Writing – review & editing. WW: Writing – original draft. YX: Methodology, Software, Writing – review & editing. XY: Writing – original draft, Writing – review & editing.

## Footnotes

^1^ http://www.ncbi.nlm.nih.gov/SNP
